# A Lower Remote Dielectric Sensing Value Was Associated with Hypovolemia and Worse Clinical Outcomes

**DOI:** 10.3390/jcm13113245

**Published:** 2024-05-31

**Authors:** Teruhiko Imamura, Toshihide Izumida, Nikhil Narang, Koichiro Kinugawa

**Affiliations:** 1 Second Department of Internal Medicine, University of Toyama, Toyama 930-0194, Japan; 2 Advocate Christ Medical Center, Oak Lawn, IL 60453, USA

**Keywords:** heart failure, congestion, diuretics

## Abstract

**Background:** Remote dielectric sensing (ReDS) systems can estimate the amount of lung fluid non-invasively and easily without expert techniques. The correlation between the elevated ReDS value and other modalities that estimate pulmonary congestion has been validated. The clinical implications of lower ReDS values, which may indicate hypovolemia, remain unknown. **Methods:** A total of 138 patients who were hospitalized for various cardiovascular-related problems and underwent ReDS value measurements at the index discharge in a blinded manner to the attending clinicians were eligible for inclusion. Patients with ReDS values > 30%, indicating the presence of pulmonary congestion, were excluded. The prognostic impact of lower ReDS values on all-cause readmission after index discharge was evaluated. **Results:** A total of 97 patients were included. The median age was 78 years, and 48 were men. The median ReDS value at index discharge was 26% (23%, 27%). A lower ReDS value correlated with smaller inferior vena cava maximum diameters (r = 0.46, *p* < 0.001) and higher blood urea nitrogen/creatinine ratios (r = −0.35, *p* < 0.001). A lower ReDS value (≤25%) was associated with a risk of all-cause readmissions with an unadjusted hazard ratio of 2.68 (95% confidence interval 1.09–6.59, *p* = 0.031) and an adjusted hazard ratio of 2.30 (95% confidence interval 0.92–5.78, *p* = 0.076). Its calculated cutoff of 25% significantly stratified the cumulative incidence of the primary outcome (36% versus 17%, *p* = 0.038). **Conclusions:** A lower ReDS value may indicate hypovolemia and be associated with the risk of all-cause readmission in patients hospitalized for cardiovascular diseases.

## 1. Introduction

Shifts between intravascular and extravascular body fluid stores are tightly regulated in healthy individuals and can be deranged in those with chronic diseases such as heart failure and kidney disease [1,2]. Such cohorts sometimes have comorbid pulmonary congestion [3]. One of the challenges of optimal congestion management is the lack of a gold standard for accurately quantifying the degree of congestion, though it remains an important surrogate marker to guide the optimization of heart failure-specific therapies to reduce the risk of readmissions [4,5,6]. Physical examination, chest X-ray, and computed tomography, together with lung ultrasound [7], are commonly used to estimate degrees of congestion. The qualitative aspects of imaging techniques require a high degree of expertise and still cannot provide a granular assessment of congestion.

Right heart catheterization is the gold standard for quantifying intracardiac pressures, though it is important to consider that intracardiac pressure measurements are not always representative of pulmonary congestion. It is also important to consider that routine application of invasive hemodynamic assessment is not practical due to resource utilization, cost, and invasiveness [8].

**Figure 1:**
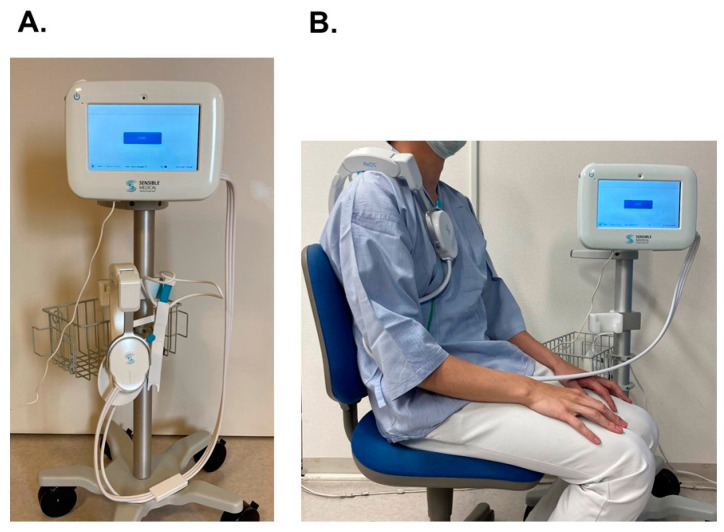
ReDS system: the ReDS device consists of a wearable sensor and monitor (**A**). A patient is asked to sit on a chair with their back against the seat while breathing naturally (**B**). The patient wears a ReDS sensor and waits for approximately 60 s. Here, the measurement does not require being naked. The ReDS value, a representative of the percentage of lung fluid, is displayed on the screen. The manufacturer-recommended normal range of the ReDS value is between 20% and 35%, although further studies are warranted to validate optimal cutoffs of normal ranges.

Remote dielectric sensing (ReDS) can quantify pulmonary congestion non-invasively without expert techniques by utilizing electromagnetic energy technology (Figure 1) [9]. The system displays the percentage of lung fluid amount as a “ReDS value”. The manufacturer proposes the normal range of ReDS value to be between 20% and 35%, although further studies are warranted to validate the proposed range [10]. The ReDS system has promise in the quantification of pulmonary congestion to guide clinicians [11]. Other modalities that estimate thoracic congestion by utilizing impedance technology have also been introduced. Of them, the ReDS system seems to have an advantage over these conventional ones in its reproducibility, easy measurement, correlation with other modalities, and prognostic impact [12,13].

The other unmet need regarding abnormal fluid balance is hypovolemia [14]. Hypovolemia can be caused by multiple external/internal triggers that may become deranged, particularly in elderly individuals. Accurate assessment of hypovolemia is often challenging. Physical examination, laboratory data, and echocardiography for assessing hypovolemia are referenced for its assessment, but there is no gold standard [15].

The ReDS system can potentially be applied for assessing the presence of hypovolemia and predicting associated worse clinical outcomes when the ReDS value is low [16,17], although there are no studies that have investigated the ability of the ReDS system to assess hypovolemia. Theoretically, a low ReDS value, probably indicating the presence of hypovolemia partially due to over-diuresis, may complicate the post-discharge care and lead to the incidence of comorbidity-related complications such as falling, especially among elderly cohorts with multiple comorbidities including cardiovascular diseases. We have aimed to evaluate the prognostic importance of low ReDS values and the correlation of these values with other clinical parameters associated with hypovolemia.

## 2. Materials and Methods

### 2.1. Patient Selection

Patients who were hospitalized for cardiovascular-related problems and received ReDS measurements at index discharge between August 2021 and February 2022 were eligible. ReDS values were measured in a blinded manner to the attending clinicians, and clinical management was performed without ReDS values. ReDS values were retrospectively reviewed for the present study after the termination of all observation periods.

Patients aged equal to or above 20 years old and who agreed to receive ReDS measurements were included. Patients exhibiting incongruent physiological parameters, such as a body mass index below 15 kg/m^2^ or exceeding 30 kg/m^2^, a body height less than 140 cm, and those with severe scoliosis, were excluded from ReDS measurements. Patients with significant pulmonary diseases, such as chronic obstructive pulmonary disease, lung cancer, and pleural effusion, were excluded, according to their impact on ReDS values due to interference tissue and sensor interference. Patients unable to maintain a seated position or tolerate the application of ReDS devices for one minute were excluded from ReDS measurements. Patients with ReDS values > 30% were excluded given the focus of this study on low ReDS values (the clinical implication of higher ReDS values, indicating the presence of pulmonary congestion, has been well studied previously) [18].

Written informed consent was obtained from all enrolled patients upon admission. The protocol received ethical approval from the local ethics committee (MTK2020007, 3 March 2021).

### 2.2. Study Outcomes

ReDS values were measured in a blinded manner, and data were prospectively collected to construct a comprehensive dataset. We performed the present study retrospectively using a prospectively constructed robust database.

ReDS values at index discharge were defined as the independent variable. All-cause readmission after index discharge was defined as the primary outcome. The primary concern was the prognostic impact of a lower ReDS value, likely indicating the presence of hypovolemia, on the primary outcome. Correlations between lower ReDS values and clinical parameters associated with hypovolemia, both of which were obtained at index discharge, were analyzed as a secondary concern.

### 2.3. ReDS Measurements

The ReDS system constitutes an advancement in non-invasive monitoring, relying on electromagnetic energy for the expeditious quantification of lung fluid levels within a minute (Figure 1) [19]. In this study, the ReDS value, a representative of lung fluid amount displayed as a percentage, was blinded to the attending clinicians, and the values were analyzed retrospectively and independently by the researchers after the completion of the observation period. Thus, clinicians managed all patients without referencing ReDS values.

This innovative system operates through the emission of low-power electromagnetic signals between two sensors seamlessly integrated into a wearable device [9]. The scrutinized signal encapsulates the dielectric properties of the lung segment situated between these sensors. Given the significantly disparate dielectric coefficients of water and air—wherein water exhibits a markedly high dielectric coefficient and air a low dielectric constant—the tissue’s dielectric coefficient is primarily governed by its fluid content. Consequently, the volumetric proportion of lung fluid (expressed as the ratio of lung fluid volume to total volume) can be meticulously computed.

ReDS values were obtained within a time frame of 45 to 60 s, with patients assuming a seated and resting position while breathing normally. The ReDS device, comprising two sonars, was positioned on the right shoulder. Subsequently, the calculated ReDS values were promptly displayed on the monitor, presented as a percentage denoting lung fluid volume relative to the total lung volume.

### 2.4. Management of Fluid Volume

Patients’ fluid volume was managed in a standard manner by referencing conventional parameters, including physical examination, chest X-ray, laboratory data, and transthoracic echocardiography [14]. As detailed above, the ReDS value was blinded to the attending clinicians, and they managed patients’ fluid volume without ReDS values. When patients were deemed to be hypovolemic, the dose of diuretics was reduced and hydration was considered, if applicable. Patients were discharged from index hospitalization after adjustment of fluid volume imbalance at the discretion of the attending clinicians.

### 2.5. Post-Discharge Management

All patients were followed at our institute or affiliated institutes by board-certified cardiologists. Patients generally came to the outpatient clinic every month, and standard examinations were performed, if applicable, including chest X-rays, laboratory tests, and transthoracic echocardiography, as well as physical examinations. Medications, including diuretics, were adjusted according to the patient’s clinical profile at the discretion of the attending clinicians.

### 2.6. Data Collection

Baseline characteristic data obtained at index discharge were retrieved, including demographic, comorbidity, laboratory, echocardiography, and medication data. ReDS values were obtained in a blinded manner to the attending clinicians and retrospectively assessed by the independent researchers. After index discharge (defined as day 0), all-cause readmission was defined as the primary outcome. At index discharge, the inferior vena cava maximum diameter was measured by transthoracic echocardiography as a secondary concern, along with the blood urea nitrogen (BUN)/creatinine (Cre) ratio.

### 2.7. Statistical Analysis

Continuous variables were expressed as medians (25% interquartile, 75% interquartile). Categorical variables were expressed as numbers and percentages. Pearson’s correlation coefficient assessed the correlation between ReDS values and clinical parameters associated with hypovolemia, both of which were obtained at index discharge as baseline characteristics, as a secondary concern.

The prognostic impact of a lower ReDS value on the primary outcome, defined as all-cause readmission, was evaluated using a time-to-event analysis performed by Cox proportional hazard ratio regression analysis. Clinically important variables that may be associated with future all-cause readmission, including ReDS values, were included in the univariable analyses, including age, body mass index, atrial fibrillation, left ventricular ejection fraction, inferior vena cava maximum diameter, systolic blood pressure, hemoglobin, serum creatinine, serum albumin, and BUN/Cre ratio. Variables with *p* < 0.05 in the univariable analyses were included in the multivariable analysis after confirming a variance inflation factor < 5.0. A receiver operating characteristic analysis was performed to calculate the cutoff of the ReDS value for the primary outcome. The cumulative incidence of the primary outcome was compared between the two groups stratified by the cutoff of the ReDS value using Kaplan–Meier analysis and the log-rank test.

## 3. Results

### 3.1. Baseline Characteristics

A total of 138 patients with ReDS values measured at index discharge were eligible. Of them, 41 patients with ReDS values > 30% were excluded, and 97 patients were included in the final cohort. All included patients were deemed to be free from significant congestion.

The median age was 78 (72, 83) years, and 48 (50%) were men (Table 1). In total, 66 (68%) had heart failure, 25 (26%) had coronary artery disease, and 46 (47%) had valvular disease. Atrial fibrillation was identified in 46 (47%) patients. The left ventricular ejection fraction was 54% (39%, 65%), and the inferior vena cava maximum diameter was 13 (12, 15) mm. The BUN/Cre ratio was 16.0 (14.0, 19.0), and the plasma B-type natriuretic peptide level was 120 (76, 251) pg/mL. Fifty-two (54%) patients received loop diuretics.

### 3.2. Correlation between ReDS Value and Clinical Parameters

**Figure 2:**
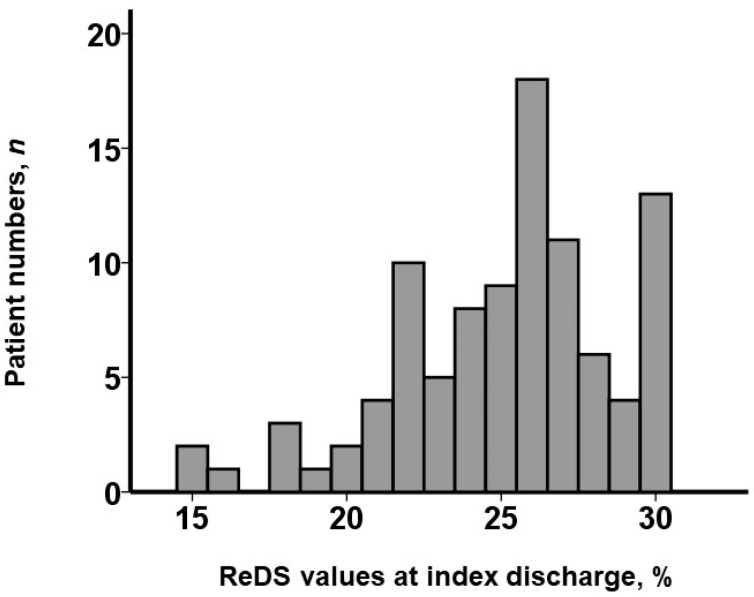
Distribution of ReDS value at index discharge: ReDS values were distributed widely, ranging between 15% and 30%. Of note, fluid volume was controlled by referencing conventional modalities, except for the ReDS value, before index discharge.

**Figure 3:**
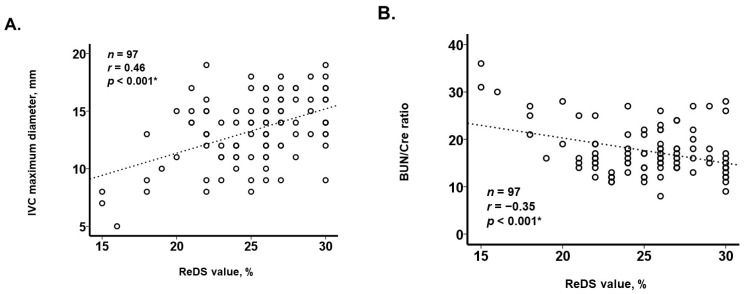
Correlation of lower ReDS values with smaller IVC maximum diameters (**A**) and with higher BUN/Cre ratios (**B**), respectively. A lower ReDS value had a mild correlation with a smaller IVC maximum diameter (**A**). A lower ReDS value had a mild correlation with a higher BUN/Cre ratio (**B**). IVC, inferior vena cava; BUN, blood urea nitrogen; Cre, creatinine. * *p* < 0.05 by Pearson’s correlation coefficient.

The ReDS value measured at index discharge in a blinded manner was distributed widely, with a median value of 26% (23%, 27%) (Figure 2). A lower ReDS value was correlated with a smaller inferior vena cava maximum diameter (r = 0.46, *p* < 0.001; Figure 3A). A lower ReDS value was also correlated with a higher BUN/Cre ratio (r = −0.35, *p* < 0.001; Figure 3B).

### 3.3. Prognostic Impact of ReDS Value

After index discharge, patients were followed for 747 (391, 838) days until their death or the termination of this study. During the observation period, 22 patients experienced a readmission, consisting of a fall, pneumonia, hypovolemia, stroke, or concern for worsening coronary artery disease or acute coronary syndrome.

Among potential variables that may be associated with the primary outcome, a lower ReDS value at index discharge, a lower left ventricular ejection fraction, and a lower systolic blood pressure were significantly associated with the primary outcome (*p* < 0.05 for all; Table 2). The inferior vena cava maximum diameter and BUN/Cre ratio did not have a significant prognostic impact in the univariable analyses. A lower ReDS value had an association with the primary outcome, though it did not reach statistical significance after multivariable analysis incorporating the above two potential confounders (adjusted hazard ratio 0.91, 95% confidence interval 0.82–1.01, *p* = 0.082).

### 3.4. Stratification of the Primary Outcome by ReDS Value

**Figure 4:**
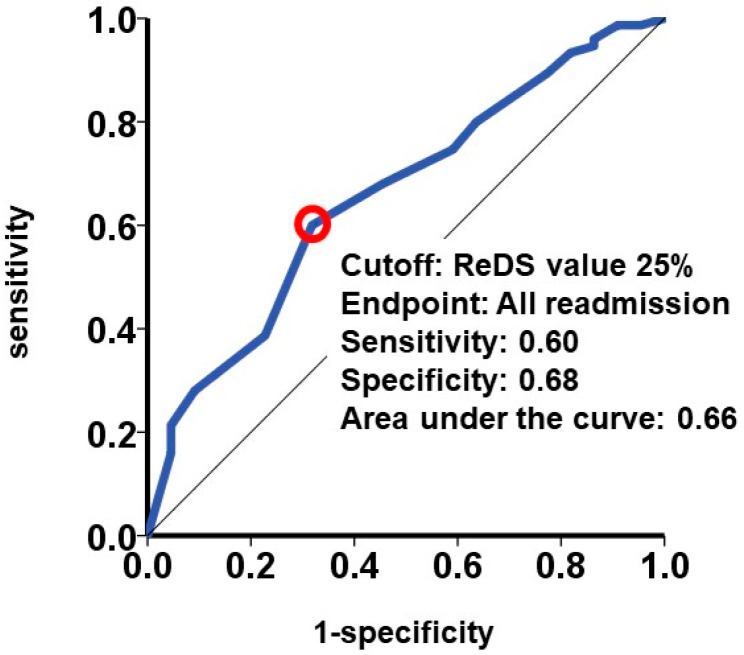
Calculation of the cutoff of the ReDS value for predicting the primary outcome. The primary outcome was defined as all-cause readmission after index discharge.

The cutoff of the ReDS value for the primary outcome was calculated as 25% with a sensitivity of 0.60, specificity of 0.68, and area under the curve of 0.66, indicating that ReDS values ≤ 25% would be significantly associated with a higher incidence of the primary outcome (Figure 4). The area under the curve for the inferior vena cava maximum diameter was 0.51 and the area under the curve for the BUN/Cre ratio was 0.53.

**Figure 5:**
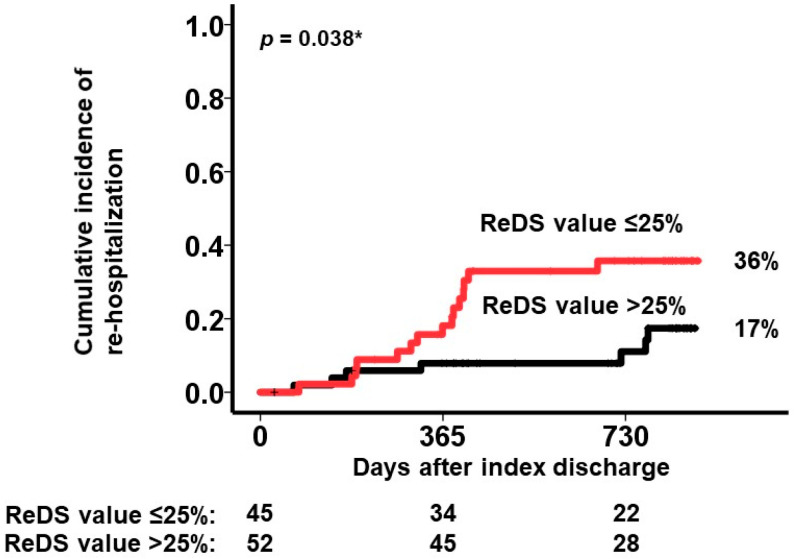
Cumulative incidence of the primary outcome stratified by the cutoff of the ReDS value at 25%. The individuals with ReDS values ≤ 25% had a significantly higher cumulative incidence of the primary outcome (red curve) compared with their counterparts (black curve). * *p* < 0.05 by log-rank test.

Forty-five patients had a ReDS value ≤ 25% at index discharge. Such patients had a significantly higher cumulative incidence of all-cause readmission (36% versus 17%, *p* = 0.038; Figure 5). A ReDS value ≤ 25% was associated with the primary outcome with an unadjusted hazard ratio of 2.68 (95% confidence interval 1.09–6.59, *p* = 0.031) and an adjusted hazard ratio of 2.30 (95% confidence interval 0.92–5.78, *p* = 0.076) by accounting for left ventricular ejection fraction and systolic blood pressure. A representative case of a patient with a ReDS value ≤ 25% is summarized in Appendix A.

## 4. Discussion

In this study, we investigated the clinical implication of low ReDS values, likely indicative of hypovolemia, in patients hospitalized for cardiovascular-related problems. ReDS values were measured blindly to the attending clinicians and were assessed retrospectively after the completion of all observation periods. Clinicians managed the patients in a standard manner without referencing ReDS values.

The main findings are as follows: ReDS values measured at index discharge were distributed widely, ranging below 20%, although the attending clinicians tried their best to correct patients’ volume status before index discharge by referencing conventional clinical parameters (without ReDS values); a lower ReDS value was correlated with a lower inferior vena cava maximum diameter; a lower ReDS value was correlated with a higher BUN/Cre ratio; and a lower ReDS value measured at index discharge was associated with all-cause readmission following index discharge with a cutoff of 25% (i.e., a lower baseline ReDS value ≤ 25% was associated with cumulative incidence of all-cause readmission).

### 4.1. Clinical Utility of ReDS System

The ReDS system has recently been introduced to quantify the lung fluid amount [16]. Recent literature demonstrated a mild to moderate correlation between the ReDS value and other conventional modalities such as chest X-ray, computed tomography, lung sonography, and B-type natriuretic peptide levels [19]. In clinical settings, the ReDS system is referenced to estimate the presence and degree of pulmonary congestion predominantly in patients with heart failure. The clinical implication of ReDS-guided management by adjusting the dose of diuretics is being studied in regard to how values trend following the adjustment of medical therapies [20].

In this study, we hypothesized that the ReDS system might also be useful for estimating the presence of hypovolemia, which is generally assessed by referencing multi-modalities but is sometimes challenging to be accurately assessed [14]. ReDS values were measured in a blinded fashion to the attending clinicians to exclude the impact of them knowing the ReDS values. If ReDS values were available to the clinicians, they could have managed patients’ volume status by referencing ReDS values (ReDS-guided management).

We excluded individuals with ReDS values > 30% (i.e., pulmonary congestion) to focus specifically on cases of low ReDS values to identify hypovolemia. We excluded individuals with obvious lung diseases and those with an extremely low/high body mass index, which may affect ReDS values [19].

### 4.2. Correlation between Low ReDS Value and Other Clinical Parameters

Hypovolemic patients present with a variety of symptoms, physical examination findings, echocardiographic findings, and laboratory abnormalities [15]. For example, physical examination reveals decreased skin turgor, low arterial blood pressure, postural hypotension, reduced jugular venous pressure, and reduced urine volume. The echocardiographic finding includes a reduction in the inferior vena cava maximum diameter [21]. Laboratory abnormalities include an elevated BUN/Cre ratio, hypernatremia or hyponatremia, hyperkalemia or hypokalemia, and metabolic alkalosis or acidosis. Recent literature recommends dynamic indices for the accurate prediction of fluid status, including stroke volume variation and pulse pressure variation, if available [22], rather than static indices such as central venous pressure [23].

However, it is sometimes challenging to accurately diagnose the presence of hypovolemia by referencing these modalities alone given the lack of gold standard, particularly in elderly patients with multiple comorbidities [24]. These parameters sometimes remain unchanged in elderly patients despite their significant hypovolemia. Sometimes we should follow the trends of these parameters to assess the presence and progression of hypovolemia, instead of just assessing the absolute value of these parameters at a certain time. For example, the clinical utility of dynamic indices, including stroke volume variation and pulse pressure variation, was proposed to assess hypovolemia in critically ill patients [22].

In this study, a lower ReDS value, probably indicating a reduced lung fluid amount, was modestly correlated with hypovolemia-related parameters such as a narrow inferior vena cava and an elevated BUN/Cre ratio, both of which are often referenced to assess the presence of hypovolemia. The ReDS system may be a practical tool to assess hypovolemia because it can repeatedly quantify the degree of hypovolemia without any expert techniques, although further studies are warranted to validate its ability to assess hypovolemia.

### 4.3. Prognostic Impact of Low ReDS Value

A lower ReDS value was associated with future all-cause readmission with a calculated cutoff of 25% (instead of the manufacturer-recommended lower limit of 20%) among the cohort, not including those with significant congestion [9]. This is not surprising because hypovolemia may cause postural dizziness, resulting in reduced daily activities of life and a higher risk of falling, especially in elderly patients [16,17]. Clinicians performed their best to adjust “clinically obvious” hypovolemia until index discharge by referencing conventional clinical parameters except for ReDS values, which were blinded to the attending clinicians. The ReDS system may be particularly useful in identifying cases of “subclinical” hypovolemia, which may in turn allow for proactive interventions to reduce the incident risk of acute kidney injury and orthostatic hypotension.

Interestingly, other hypovolemia-related parameters such as the BUN/Cre ratio were not associated with the primary outcome. The changes in these conventional parameters may be trivial in the case of subclinical hypovolemia, particularly in the elderly cohort. Again, clinically obvious hypovolemia should have been corrected by the attending clinicians before index discharge.

### 4.4. Clinical Implications of Our Findings

Clinical diagnosis of hypovolemia is often challenging, particularly in elderly patients with multiple comorbidities, because hypovolemia-related changes in clinical parameters are often trivial in these cohorts. In this context, the ReDS system may be a promising tool for quantifying the degree of hypovolemia and risk stratification for future events. The ReDS system could be useful, particularly in identifying subclinical hypovolemia, which is challenging to identify by using conventional modalities alone. Clinically obvious hypovolemia often presents typical clinical signs and symptoms, which let us assess the presence of hypovolemia and manage them. We do not deny the clinical utility of conventional modalities to assess hypovolemia. We usually reference these conventional modalities in combination to assess the presence and degree of hypovolemia. The ReDS system might have an additive impact in assessing the presence and degree of hypovolemia upon these conventional modalities when used together. Further prospective multi-center studies are warranted to validate the clinical implication of ReDS-guided hypovolemia management.

### 4.5. Limitations

The current study has several potential limitations. The sample size was moderate. Given the small event number, we could not construct a robust multivariable model for predicting the primary outcome consisting of statistically significant variables. Of note, the prognostic impact of ReDS values did not reach statistically significant levels in the multivariable analysis. ReDS values had a moderate correlation with several hypovolemia-related clinical parameters. Given the lack of a gold standard for quantifying hypovolemia, it may not be surprising that ReDS values did not have a strong correlation with them. We excluded several patients for whom ReDS measurements were not recommended by the manufacturer, such as those with BMI > 30. The applicability of the ReDS system in such excluded cohorts requires further validation studies. Clinicians tried their best to adjust participants’ hypovolemia by referencing conventional clinical parameters, except for ReDS values, and our cohort may not include individuals with clinically obvious and severe hypovolemia (such clinically obvious severe hypovolemia should have been correctly treated before index discharge). Nevertheless, a lower ReDS value, probably indicating subclinical hypovolemia in this study, was associated with worse clinical outcomes. We measured ReDS values only once at index discharge, and the clinical implications of the trend of ReDS values remain unknown.

## 5. Conclusions

A lower ReDS value may indicate hypovolemia and be associated with the risk of all-cause readmission in patients hospitalized for cardiovascular diseases. Further prospective multi-center studies are warranted to validate our findings in a variety of clinical scenarios. The clinical implication of the ReDS-guided management of hypovolemia remains the next concern that should be studied.

## Figures and Tables

**Table 1:** Baseline characteristics at index discharge.

	*N* = 97
Demographics	
Age, years	78 (72, 83)
Men	48 (50%)
Body mass index, kg/m^2^	21.3 (19.4, 24.8)
Hemodynamics	
Systolic blood pressure, mmHg	106 (95, 120)
Pulse rate, bpm	69 (64, 75)
Comorbidity	
Heart failure	66 (68%)
Hypertension	79 (81%)
Dyslipidemia	56 (58%)
Diabetes mellitus	31 (32%)
Atrial fibrillation	46 (47%)
History of stroke	11 (11%)
Coronary artery disease	25 (26%)
Valvular disease	46 (47%)
Echocardiography	
Left ventricular end-diastolic diameter, mm	51 (46, 60)
Left ventricular ejection fraction, %	54 (39, 65)
Left atrial diameter, mm	40 (37, 48)
Inferior vena cava maximum diameter, mm	13 (12, 15)
Laboratory data	
Hemoglobin, g/dL	12.3 (11.2, 13.3)
Serum albumin, g/dL	3.5 (3.2, 3.8)
Serum sodium, mEq/L	139 (137, 141)
Serum Cre, mg/dL	0.93 (0.81, 1.41)
BUN/Cre ratio	16.0 (14.0, 19.0)
Plasma B-type natriuretic peptide, pg/mL	120 (76, 251)
Medications	
Beta-blocker	73 (75%)
Renin–angiotensin system inhibitor	89 (92%)
Mineralcorticoid receptor antagonist	43 (44%)
Sodium–glucose cotransporter inhibitor	38 (39%)
Loop diuretics	52 (54%)
Vasopressin type 2 receptor antagonist	28 (29%)

Baseline characteristic data at index discharge are summarized. BUN, blood urea nitrogen; Cre, creatinine. Continuous variables are stated as medians (25% interquartile, 75% interquartile) and categorical variables are stated as numbers and percentages.

**Table 2:** Variables associated with future all-cause readmission.

	Univariable Analysis		Multivariable Analysis	
	Hazard Ratio (95% CI)	*p*-Value	Hazard Ratio (95% CI)	*p*-Value
Age, years	0.98 (0.95–1.01)	0.16		
Body mass index, kg/m^2^	1.00 (0.99–1.01)	0.81		
Atrial fibrillation	1.65 (0.70–3.85)	0.25		
LVEF, %	0.98 (0.95–0.99)	0.044 *	0.98 (0.96–1.01)	0.12
IVC maximum diameter, mm	0.99 (0.86–1.16)	0.36		
Systolic blood pressure, mmHg	0.97 (0.95–1.00)	0.048 *	0.98 (0.95–1.01)	0.10
Hemoglobin, g/dL	1.08 (0.85–1.38)	0.52		
Serum Cre, mg/dL	0.99 (0.66–1.50)	0.98		
Serum albumin, g/dL	0.37 (0.09–1.58)	0.18		
BUN/Cre ratio	1.03 (0.96–1.11)	0.47		
ReDS value at index discharge, %	0.89 (0.81–0.99)	0.032 *	0.91 (0.82–1.01)	0.082

Baseline characteristics that were potentially deemed to be associated with the primary outcome were included in the univariable analyses. Variables with *p* < 0.05 in the univariable analyses were included in the multivariable analysis. CI, confidence interval; LVEF, left ventricular ejection fraction; IVC, inferior vena cava; Cre, creatinine; BUN, blood urea nitrogen; ReDS, remote dielectric sensing. * *p* < 0.05 by Cox proportional hazard ratio regression analysis.

## Data Availability

Data are available from the corresponding author upon reasonable request.

## Appendix A. Representative Case Presentation

A 77-year-old woman was hospitalized for suspected aortic stenosis. At index discharge, she did not have any symptoms or signs that indicated the presence of hypovolemia. Serum BUN was 31.9 mg/dL, serum Cre was 1.20, the BUN/Cre ratio was 26.6, serum albumin was 3.0 g/dL, and hemoglobin was 11.9 g/dL. The left ventricular end-diastolic diameter was 46 mm, the left ventricular ejection fraction was 71%, and the inferior vena cava maximum diameter was 13 mm. Blood pressure was 104/64 mmHg and pulse rate was 85 bpm. She was diagnosed with moderate aortic stenosis and decided to be medically followed up with scheduled echocardiography. At 191 days following index discharge, she was readmitted due to falling.

The ReDS value was measured blindly at index discharge. After the completion of the observation period, the ReDS value at index discharge was clarified as 22% (<25%).
